# Ascending aortic dilation in adult patients with congenital ventricular septal defect: An observational study

**DOI:** 10.1097/MD.0000000000010383

**Published:** 2018-04-13

**Authors:** Hai-Yang Li, Yuan-Fei Zhao, Lu Dai, Shi-Jun Xu, Hong-Jia Zhang, Wen-Jian Jiang

**Affiliations:** a Department of Cardiac Surgery, Beijing Anzhen Hospital, Capital Medical University, Beijing, China; b Centre for Transplant and Renal Research, The Westmead Institute for Medical Research, University of Sydney, Sydney, Australia.; c Beijing Institute of Heart, Lung and Blood Vessel Diseases; d Beijing Lab for Cardiovascular Precision Medicine; e Beijing Aortic Disease Center, Cardiovascular Surgery Center; f Beijing Engineering Research Center for Vascular Prostheses, Beijing, China.

**Keywords:** adult patient, ascending aortic dilation, clinical features, congenital ventricular septal defect, surgical management

## Abstract

Many adult patients with congenital ventricular septal defect (VSD) also developed ascending aortic dilation, but few report the clinical features and surgical management of these patients. This study was designed to study ascending aortic dilation in adult patients with congenital VSD, and summarized the treatment experience and prognosis.

To assess the clinical features and surgical management, we performed a retrospective analysis on preoperative data, intraoperative data, and postoperative data from the adult patients with congenital VSD who developed ascending aortic dilation in our institution from February 2010 to December 2016.

From February 2010 to December 2016, we operated on 13 adult patients (12 males, 92.31%) with VSD who developed ascending aortic dilation. Median age was 37 (interquartile range 14) years. All patients suffered from perimembranous VSD and received surgical treatment. Their symptoms were all improved after surgery, no deaths occurred.

Surgery is feasible for the ascending aortic dilation in adult patients with congenital VSD. Both proper perioperative treatment and close monitoring are required for the successful surgery.

## 1 Introduction

Some adult patients with congenital heart disease simultaneously developed aortic disease, including aortic valve disease, aortic dissection, and ascending aortic dilation.^[1–3]^ There are many studies about the ascending aortic dilation with tetralogy of Fallot (TFO), truncus arteriosus, or transposition of the great arteries (TGA), but few report the ascending aortic dilation with congenital ventricular septal defect (VSD).^[4–7]^ According to our experience, there were 0.89% (21–2347) adult patients with VSD among all those suffered from ascending aortic dilation. Owing to the anatomic relationship between the VSD and aortic structure, there might be some potential reoperations of aortic disease after VSD repair. So it was extremely vital to address aortic disorder in patients with congenital VSD.^[7,8]^ However, up-to-date little data are available in literature with respect to clinical features and surgical management of ascending aortic dilation with congenital VSD.

To address this issue, we performed a retrospective analysis of the clinical data of 13 cases of ascending aortic dilation with VSD whom were operated on from February 2010 to December 2016 at our center. In analyzing of clinical characteristics and treatment, we hope this study could help surgeons to manage ascending aortic dilation with congenital VSD.

## 2 Patients and methods

### 2.1 Patients

To assess the clinical features and surgical management, we performed a retrospective analysis on preoperative data, intraoperative data, and postoperative data from the adult patients with VSD who developed ascending aortic dilation at our center from February 2010 to December 2016. The patients, who underwent VSD repair before the aortic surgery, were excluded from this study because the present study focused on adult patients with ascending aortic dilation underwent aortic and VSD repair at the same time. The treatment to the patients who underwent VSD repair before the aortic surgery was different from that in the present study. Before the surgical treatment, computed tomographic angiography (CTA), chest X-ray, electrocardiogram, transthoracic echocardiography, and all routine inspections were performed in each case. There was no obvious specificity for the diagnosis of ascending aortic dilation and VSD by chest X-ray and electrocardiogram. So the ascending aortic disorder, VSD location, and other complications would be shown by CTA and echocardiography. This study was approved by Beijing Anzhen Hospital Ethical Committee and all research methods in this study were in the accordance with the approved guidelines. All patients provided informed written consent.

### 2.2 Surgical techniques and approaches

Under general anesthesia, all patients received midsternotomy. After systemic heparinization, the regular cardiopulmonary bypass (CPB) was established with inferior and superior vena cava cannulation, 7 patients with right femoral arterial cannulation, and 6 patients with ascending aortic arterial cannulation because their distal ascending aorta were normal. All the left heart drainages were done throng the right superior pulmonary vein. After aortic clamp, antegrade ardioplegia perfusion was performed. After that, the VSD was closed through right atrium and interatrial septum, including 4 closures with pericardium and 9 closures with direct suture. During the surgery, 1 patient under atrial septal defect (ASD) closure and 1 patient underwent patent ductus arteriosus (PDA) closure. Finally, 10 patients underwent Bentall procedure and 3 patients underwent the prosthetic ascending aorta replacement as previously reported.^[9–11]^

### 2.3 Patient follow-up

All patients were monitored by clinic visits or phone calls, and by the referring physician to document survival and adverse events. Patients were recommended to have an echocardiography annually to evaluate the surgical outcome and detect complications. Follow-up was complete in all patients, with the median 30.56 (interquartile range [IQR] 55) months.

### 2.4 Statistical analysis

Data were analyzed using Prism 5.01 for Windows (GraphPad Software, Inc., La Jolla, CA). Descriptive statistics for categorical variables are reported as frequency and percentage; continuous variables are reported as mean ± SD or median (IQR) appropriate. Nonparametric test was used to detect differences between groups. Differences were considered to be statistically significant at a *P* value of <.05

## 3 Results

### 3.1 Preoperative characteristics

From February 2010 to December 2016, 2347 adult patients with ascending aortic dilation received aortic surgery in our hospital. There were 0.89% (21–2347) adult patients with VSD among all those suffered from ascending aortic dilation. The present study focused on patients with ascending aortic dilation underwent aortic and VSD closure at the same time. So 13 patients (12 males, 92.31%) were included in this study, with the median 37 (IQR 14) years. Six patients had chest tightness and shortness of breath, whereas the other 7 patients were asymptomatic and diagnosed by routine examination. There were 3 bicuspid aortic valves (23.07%) and 1 Marfan syndrome (7.69%). No patient in this study suffered rheumatism, endocarditis, or diabetes. The mean preoperative systolic blood pressure was 133.46 ± 24.57 mm Hg and diastolic blood pressure was 68.31 ± 6.81 mm Hg. The mean preoperative creatinine, oxygen saturation, arterial partial pressure of oxygen, and cardiothoracic ratio were 69.75 ± 14.43 μmol/L, 96.89 ± 1.35%, 86.83 ± 7.52 mm Hg, and 0.54 ± 0.09. One patient combined with ASD and another one with PDA simultaneously (Table 1).

**Table 1:**
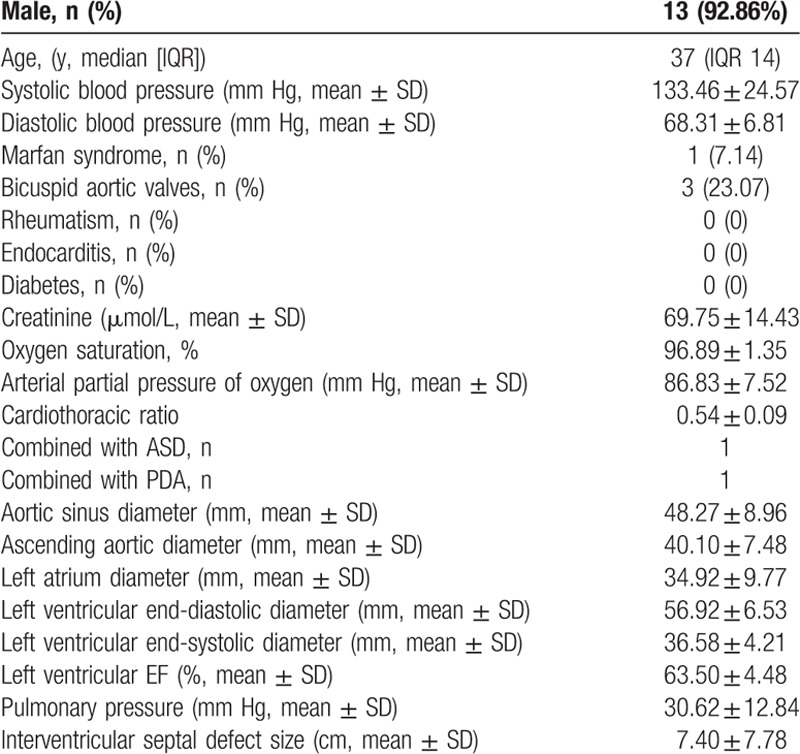
Patients’ profiles.

The transthoracic echocardiography showed that all the patients suffered from perimembranous VSD. The moderate or severe aortic insufficiency could be detected in 10 patients. The mean aortic sinus diameter calibrated by preoperative CTA was 48.27 ± 8.96 mm and the mean ascending aortic diameter was 40.10 ± 7.48 mm. The mean interventricular septal defect size calibrated by preoperative echocardiography was 7.40 ± 7.78 cm, mean left atrium diameter was 34.92 ± 9.77 mm, mean left ventricular end-diastolic diameter (LVEDD) was 56.92 ± 6.53 mm, mean left ventricular end systolic diameter was 36.58 ± 4.21 mm, and mean left ventricular ejection fraction (LVEF) was 63.50 ± 4.48%. The mean pulmonary pressure calibrated by preoperative echocardiography was 30.62 ± 12.84 mm Hg (Table 1).

### 3.2 Surgical strategy and intraoperative data

All the patients received VSD closure; 10 patients with moderate or severe aortic insufficiency received Bentall procedure at the same time, whereas the other 3 patients received ascending aortic replacement. Four patients received VSD closure with pericardium, 9 patients with derect suture. One patient received ASD repair, and 1 patient received PDA closure at the same time. The CPB time was 150.54 ± 49.33 minutes, and the aortic cross-clamp time was 97.46 ± 39.65 minutes. No patient underwent repeat sternotomy due to bleeding (Table 2).

**Table 2:**
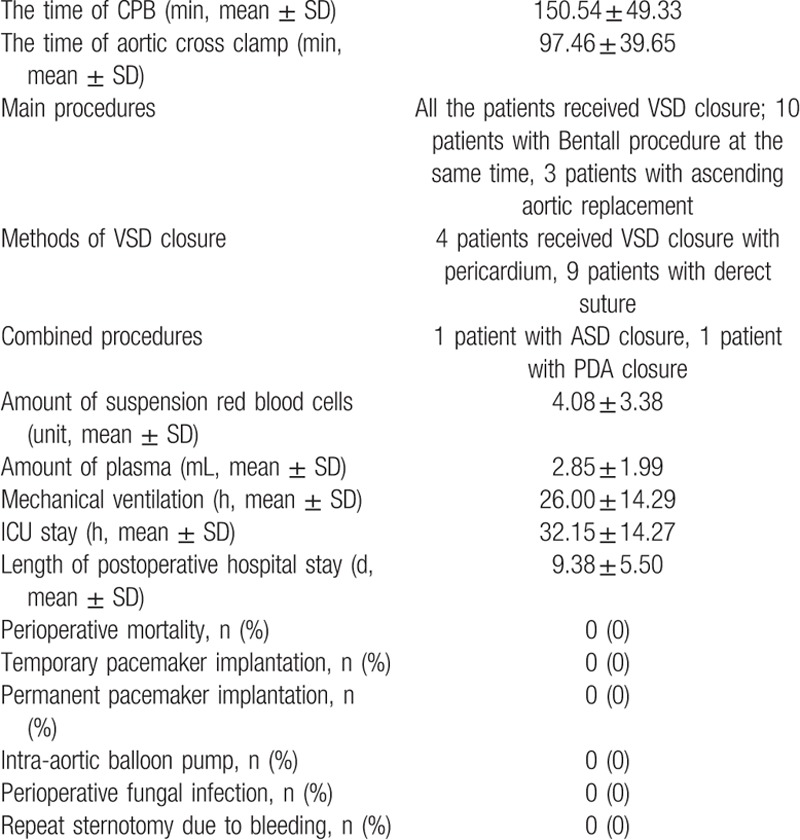
Surgical strategy and intraoperative data.

After the surgery, the length of ICU stay was 32.15 ± 14.27 hours and the length of postoperative hospital stay was 9.38 ± 5.50 days. No patient received temporary or permanent pacemaker, and intra-aortic balloon pump implantation. There was no perioperative fungal infection (Table 2).

### 3.3 Follow-up

Follow-up was complete in all patients, with the median 30.56 (IQR 55) months, no stroke and acute renal failure. Echocardiography showed no abnormality during follow-up; all the patients lived well (Table 3).

**Table 3:**
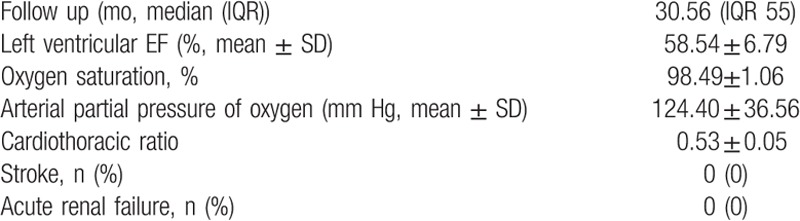
Follow-up.

The mean LVEF calibrated by preoperative echocardiography was 58.54 ± 6.79%, which was similar (*P* = .0837) to the preoperative value. Compared with the preoperative value, both postoperative oxygen saturation (98.49 ± 1.06% vs 96.89 ± 1.35%, *P* = .0251) and arterial partial pressure of oxygen (124.40 ± 36.56 mm Hg vs 86.83 ± 7.52 mm Hg, *P* = .0100) improved significantly. The postoperative cardiothoracic ratio was 0.53 ± 0.05, which was similar (*P* = .6629) to the preoperative value (Table 3).

## 4 Discussions

A number of adult patients with congenital heart disease will go on to develop progressive aortic dilation^[1–3]^; however, the risk of the attendant complications in any individual patient remains unknown.^[12]^ The outcomes of interest in follow-up of dilated ascending aorta are rupture, aortic dissection, and death.^[13]^ To avoid the complications described above, severe ascending aortic dilation should be treated by the replacement of ascending aorta or remodeling/replacement of aortic root, which are similar to the simple ascending aortic dilation.^[14,15]^

As we all know, many ascending aortic dilations are asymptomatic when diagnosed, being incidentally noted on chest X-ray or echocardiographic evaluation of aortic insufficiency.^[16]^ However, about 25% to 75% of ascending aortic dilatation patients present with anterior chest pain.^[16–18]^ The pain may be acute in onset signifying impending rupture, or a chronic gnawing pain from compression of the overlying sternum. Signs of superior vena caval or airway compression are present when the ascending aortic aneurysms are so large.^[18]^ There are some specific clinical manifestations for patients with VSD. The patients with small VSD are asymptomatic and diagnosed because of a loud systolic murmur, prompting an echocardiogram.^[19]^ Some adult patients with large VSD present with tachypnea and diaphoresis with activity and are at risk for repeated upper respiratory tract infections and cardiac asthma.^[20]^ In this study, 6 patients had chest tightness and shortness of breath, whereas the other 7 patients were asymptomatic and diagnosed by routine examination.

In addition to physical symptoms, echocardiography also has diagnostic value for ascending aortic dilation and VSD. Ascending aortic dilations are the most common cause of isolated aortic insufficiency, and therefore aneurysms are frequently detected during evaluation of a regurgitant aortic valve.^[21]^ However, transthoracic echocardiography is far less reliable to diagnose ascending aortic dilation.^[17]^ Echocardiography evaluation is the most widely used imaging to diagnose and characterize a VSD. To assess a VSD completely, one must not only localize it but also define its shape and dimensions, which is accomplished by viewing the defect from multiple imaging planes.^[19]^ All the VSDs in this study were perimembranous and the mean size of VSDs was 7.40 ± 7.78 cm^2^. During diagnosis, CTA is another important tool, which provides rapid and precise evaluation of the ascending aorta but less value for the VSD. More accurate assessment of aneurysm size can be shown in CTA than in echocardiography.^[19]^

When ascending aortic dilation in adult patient with congenital VSD was diagnosed, surgical treatment is the only effective treatment. For all the patients, we used the right femoral artery, superior and inferior vena caval to establish cardiopulmonary bypass. For all VSDs in this study, right atrial incision and atrial septal incision have good exposure. Eight patients (61.54%) received the direct closure of VSD and the others with patch.

Both proper perioperative treatment and close monitoring are required and significant to ensure a successful operation and good surgical recovery. For ascending aortic dilation in patients with congenital VSD, in addition to regular postoperative care, the amount of liquid and the heart function should be monitored carefully. Acute heart failure and pulmonary edema were very common after VSD closure, so cardiotonics and diuretics for these patients. In addition, considering the longtime of aortic cross clamp for ascending aortic and VSD surgical procedure, more attention should be paid to the recovery of consciousness, change of ventilation/perfusion ratio, and temperature of limbs and muscle tension. The major cause of perioperative death of VSD closure was the acute failure of right heart. The perioperative mortality rate of VSD was between 0% and 3.7%,^[22,23]^ whereas the ascending aortic aneurysm was about 4.2%,^[24]^ but there is no published data for ascending aortic dilation in patients with congenital VSD. Operation effect of ascending aortic dilation in patients with congenital VSD is usually good. There is no perioperative death in this study and all the patients survived after the surgery with the median 30.56 (IQR 55) months follow-up.

## 5 Study limitations

One of the major limitations of this study was the small number of patients. Another significant limitation of this study postoperative data of the present study was not enough, and the long-term follow-up result was still needed.

## 6 Conclusions

Our study suggests that surgery is feasible for the ascending aortic dilation in adult patients with congenital VSD, whereas early diagnosis and timely surgical treatment are the key bases for the successful surgery. Besides, both proper perioperative treatment and close monitoring are required and significant to ensure a successful operation and good surgical recovery.

## Author contributions

**Conceptualization:** Hai-Yang Li, Yuan-Fei Zhao, Hongjia Zhang, Wen-Jian Jiang.

**Data curation:** Hai-Yang Li, Lu Dai, Wen-Jian Jiang.

**Formal analysis:** Yuan-Fei Zhao, Lu Dai.

**Funding acquisition:** Hongjia Zhang.

**Investigation:** Lu Dai, Shi-Jun Xu, Wen-Jian Jiang.

**Methodology:** Lu Dai, Shi-Jun Xu.

**Project administration:** Hongjia Zhang.

**Supervision:** Yuan-Fei Zhao, Hongjia Zhang, Wen-Jian Jiang.

**Writing – original draft:** Hai-Yang Li, Yuan-Fei Zhao, Hongjia Zhang, Wen-Jian Jiang.

**Writing – review & editing:** Yuan-Fei Zhao, Hongjia Zhang, Wen-Jian Jiang.
